# Rapid short-duration hypothermia with cold saline and endovascular cooling before reperfusion reduces microvascular obstruction and myocardial infarct size

**DOI:** 10.1186/1471-2261-8-7

**Published:** 2008-04-10

**Authors:** Matthias Götberg, Goran K Olivecrona, Henrik Engblom, Martin Ugander, Jesper van der Pals, Einar Heiberg, Håkan Arheden, David Erlinge

**Affiliations:** 1 Department of Cardiology, Lund University Hospital, 221 85, Lund, Sweden; 2 Department of Clinical Physiology, Lund University Hospital, 221 85, Lund, Sweden

## Abstract

**Background:**

The aim of this study was to evaluate the combination of a rapid intravenous infusion of cold saline and endovascular hypothermia in a closed chest pig infarct model.

**Methods:**

Pigs were randomized to pre-reperfusion hypothermia (n = 7), post-reperfusion hypothermia (n = 7) or normothermia (n = 5). A percutaneous coronary intervention balloon was inflated in the left anterior descending artery for 40 min. Hypothermia was started after 25 min of ischemia or immediately after reperfusion by infusion of 1000 ml of 4°C saline and endovascular hypothermia. Area at risk was evaluated by in vivo SPECT. Infarct size was evaluated by ex vivo MRI.

**Results:**

Pre-reperfusion hypothermia reduced infarct size/area at risk by 43% (46 ± 8%) compared to post-reperfusion hypothermia (80 ± 6%, p < 0.05) and by 39% compared to normothermia (75 ± 5%, p < 0.05). Pre-reperfusion hypothermia infarctions were patchier in appearance with scattered islands of viable myocardium. Pre-reperfusion hypothermia abolished (0%, p < 0.001), and post-reperfusion hypothermia significantly reduced microvascular obstruction (10.3 ± 5%; p < 0.05), compared to normothermia: (30.2 ± 5%).

**Conclusion:**

Rapid hypothermia with cold saline and endovascular cooling before reperfusion reduces myocardial infarct size and microvascular obstruction. A novel finding is that hypothermia at the onset of reperfusion reduces microvascular obstruction without reducing myocardial infarct size. Intravenous administration of cold saline combined with endovascular hypothermia provides a method for a rapid induction of hypothermia suggesting a potential clinical application.

## Background

Modern reperfusion therapy of acute myocardial infarction is aimed at performing primary percutaneous coronary intervention (PCI) in order to reduce myocardial infarct size and the extent of complications [1-3]. Although restoration of blood flow to the jeopardized myocardium is a prerequisite for myocardial salvage, reperfusion in itself may lead to accelerated and additional myocardial injury beyond that generated by ischemia alone, a phenomenon referred to as "reperfusion injury" [4,5]. Furthermore, myocardial ischemia damages the endothelium causing impairment of the microvascular blood flow (microvascular obstruction). In the clinical setting, microvascular obstruction is common and associated with a worse clinical outcome. [6]

Consequently, there is a need for an adjunctive therapy for myocardial salvage beyond that which modern reperfusion therapy can provide. There are several experimental pharmacological therapies which have shown to protect the myocardium from ischemic injury; however for various reasons, no therapy has yet reached clinical practice [7]. Hypothermia as a possible therapeutic option in treating myocardial infarction has in experimental studies shown beneficial results [8-14]. Furthermore, Hale et al have demonstrated that hypothermia reduces the extent of microvascular obstruction [15]. However, local cooling just before reperfusion did not reduce infarct size [10]. Two randomized clinical trials investigating the effects of hypothermia as an adjunct therapy to PCI in patients with acute myocardial infarction failed to show positive results [16,17]. However, post hoc analysis of the patients who reached a temperature of < 35°C before reperfusion showed a reduction in infarct size suggesting the benefit of induction of hypothermia before reperfusion. Due to the large body mass of humans in the clinical setting it is difficult to achieve adequate hypothermia without delaying reperfusion therapy. With external cooling or endovascular cooling alone it takes 30 min to 1 h for the patients to reach target temperature [8,18-20]. The aim of this study was to investigate whether rapid induction of hypothermia before reperfusion (pre-reperfusion hypothermia) would reduce infarct size in a closed chest pig model. We also wanted to test this hypothesis with a clinically applicable treatment protocol that would achieve a rapid cooling to target temperature in less than 15 minutes. We therefore combined an infusion of cold saline together with endovascular cooling in order to achieve rapid cooling and compared it with hypothermia induced immediately after reperfusion (post-reperfusion hypothermia) or normothermia. Based on the results from previous clinical trials our hypothesis was that hypothermia had to be induced before reperfusion in order to reduce myocardial injury.

## Methods

### Ethics

The study conforms to the Guide for the Care and Use of Laboratory Animals, US National Institute of Health (NIH Publication No. 85–23, revised 1996) and was approved by the Ethics Committee of Lund University, Sweden.

### Experimental preparation

22 healthy domestic male and female pigs weighing 40–50 kg were fasted overnight with free access to water and were premedicated with Ketaminol (Ketamine, Intervet, Danderyd, Sweden), 100 mg/ml, 1,5 ml/10 kg, and Rompun (Xylazin, Bayer AG, Leverkusen, Germany), 20 mg/ml, 1 ml/10 kg intramuscularly 30 min before the procedure. After induction of anesthesia with thiopental 12.5 mg/kg (Pentothal, Abbott, Stockholm, Sweden), the animals were orally intubated with cuffed endotracheal tubes. A slow infusion of 1 μ l/ml Fentanyl (Fentanyl, Pharmalink AB, Stockholm, Sweden) in buffered glucose (25 mg/ml) was started at a rate of 2 ml/min and adjusted as needed. Anesthesia was complemented with small intermittent doses of thiopental (Pentothal, Abbott, Stockholm, Sweden), 50 mg/ml, 1–2 ml when needed. Mechanical ventilation was established with a Siemens-Elema 900B ventilator in the volume-controlled mode, adjusted in order to obtain normocapnia (pCO2: 5.0–6.0 kPa). The animals were ventilated with a mixture of nitrous oxide (70%) and oxygen (30%). Analysis of arterial bloodgases in order to adjust ventilation was performed before initiation of ischemia, and once during ischemia. The pigs were continuously monitored with electrocardiogram (ECG). Heparin (200 IU/kg) was given intravenously at the start of the catheterization. A 12 F introducer sheath (Boston Scientific Scimed, Maple Grove, MN, USA) was inserted into the surgically exposed left femoral vein. Through the introducer a 0.021-inch guide wire (Safe-T-J Curved™, Cook Medical Inc, Bloomington, IN, USA) was inserted into the proximal inferior vena cava. Using the guide wire, a 10.7 F Celsius Control™ catheter (Innercool Therapies Inc, San Diego, CA, USA) was then placed into the inferior vena cava with the tip of the catheter at the level of the diaphragm. Body temperature was measured with a temperature probe (TYCO Healthcare Norden AB, Solna, Sweden) placed in the distal part of the esophagus. The catheter and the temperature probe were then connected to the Celsius Control and the system was set to maintain a normal pig body temperature of 38.0°C. A 6 F introducer sheath (Boston Scientific Scimed, Maple Grove, MN, USA) was then inserted into the surgically exposed left carotid artery upon which a 6 F JL4 Wiseguide™ (Boston Scientific Scimed, Maple Grove, MN, USA) was inserted into the left main coronary artery. The catheter was used to place a 0.014-inch PT Choice™ guide wire (Boston Scientific Scimed, Maple Grove, MN, USA) into the distal portion of the LAD. A 3.0 × 20 mm Maverick monorail™ angioplasty balloon (Boston Scientific Scimed, Maple Grove, MN, USA) was then positioned in the mid portion of the LAD, immediately distal to the first diagonal branch. All radiological procedures were performed in an experimental catheterization laboratory (Shimadzu Corp., Kyoto, Japan).

### Ischemia protocol

After a stable core body temperature of 38.0°C was achieved, ischemia was induced by inflation of the angioplasty balloon for 40 min. An angiogram was performed after inflation of the balloon and before deflation of the balloon in order to verify total occlusion of the coronary vessel and correct balloon positioning. After deflation of the balloon a subsequent angiogram was performed to verify restoration of blood flow in the previously occluded artery.

### Hypothermia protocol

**Figure 1:**
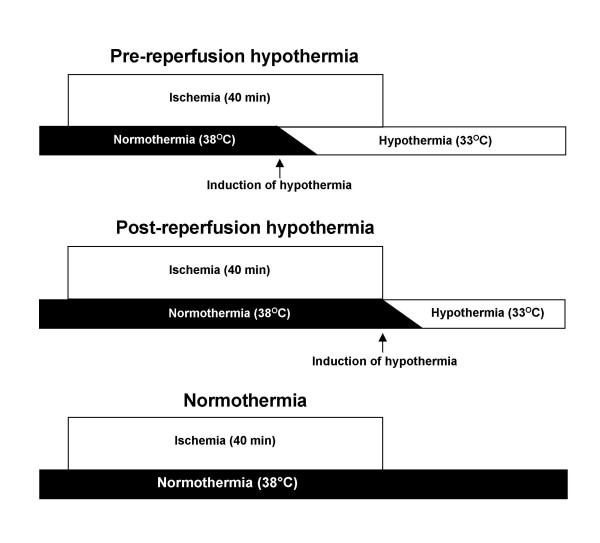
**Protocol for induction of hypothermia.** In the pre-reperfusion group, hypothermia was started after 25 min of ischemia (15 min before reperfusion) and in the post-reperfusion group, hypothermia was started immediately after reperfusion. The normothermic group was maintained at 38.0°C.

The pigs were randomized by drawing folded paper notes out of a box to rapid hypothermia before reperfusion, (pre-reperfusion hypothermia, n = 8) or immediately after reperfusion (post-reperfusion hypothermia, n = 8). A normothermic group (n = 6) was also studied in order to provide comparison between different hypothermia protocols and normothermia. Hypothermia was induced by a rapid intravenous infusion of 1000 ml of 4°C cold saline into a central vein together with the Celsius Control™ endovascular cooling system after 25 min of ischemia or immediately after reperfusion when coronary blood flow was restored (Figure 1). Target temperature was 33°C and successful cooling was defined as a temperature of = 35°C. Hypothermia was then actively maintained for 30 min followed by passive rewarming with blankets.

### In vivo assessment of area at risk by SPECT

Single photon emission computed tomography (SPECT) was used to assess the area at risk (AAR). Five hundred MBq of ^99m^Tc-tetrofosmin was administered intravenously ten minutes before deflation of the angioplasty balloon. The anesthetized pigs were then imaged in a supine position with a dual head camera (ADAC Vertex, Milpitas, CA, USA) at 32 projections (40 s per projection) with a 64 × 64 matrix yielding a digital resolution of 5 × 5 × 5 mm. Iterative reconstruction using maximum likelihood-expectation maximization (MLEM) was performed with a low-resolution Butterworth filter with a cut-off frequency set to 0.6 of Nyquist and order 5.0. No attenuation or scatter correction was applied. Finally short and long-axis images were reconstructed. Quantification of the size of AAR in ml was performed automatically as the extent of the perfusion defect as determined by commercially available software (Auto QUANT™ 4.3.1 and a standard database; ADAC, Milpitas, CA, USA) [21]. AAR was expressed as percent of the left ventricular volume, and this was determined by dividing the AAR (ml) from SPECT by the left ventricular wall volume (ml) determined by ex vivo MRI as described below. This was performed due to the known limitations in accuracy for determining left ventricular wall volume by SPECT [22].

### In-vivo measurement of cardiac output and stroke volume

Cardiac output was assessed by magnetic resonance flow imaging of a cross section of the pulmonary trunk using a velocity encoded gradient echo sequence with retrospective ECG triggering. Typical imaging parameters were: slice thickness 6 mm, number of time frames per cardiac cycle 35, echo time 5.6 ms, repetition time 9.0 ms, spatial resolution 1.5 × 1.5 × 8 mm, velocity encoding gradient (VENC) 200 cm/s, flip angle 15°. Image analysis for flow quantification was performed according to established techniques [23] using freely available software Segment 1.611 [24].

### Infarct size and microvascular obstruction assessed by ex vivo MRI

*Ex vivo *imaging of the heart was undertaken using a 1.5 T Philips Intera CV MR scanner (Philips, Best, the Netherlands) according to a previous described protocol [25]. In brief, a commercially available gadolinium-based contrast agent (Magnevist, *gadopentetate dimeglumine*, Gd-DTPA, Schering Nordisker AB, Järfälla, Sweden) was administered intravenously (0.2 mmol/kg) both 60 and 15 minutes prior to removal of the heart. After removal, the heart was immediately rinsed in cold saline and the ventricles were filled with balloons containing deuterated water. Three dimensional acquisition of T1-weighted images (TR = 20 ms, TE = 3.2 ms, flip angle = 70° and 2 averages) yielded a stack of approximately 200 images with an isometric resolution of 0.5 mm covering the entire heart. Images were then acquired using a head coil and the duration of acquisition was typically 45 minutes.

The MR images were analyzed using freely available software [26]. The endocardial and epicardial borders of the left ventricular myocardium were manually delineated in short-axis *ex vivo *images. This defined the volume of left ventricular myocardium (cm^3 ^= ml). The infarct size (IS) was first determined as the volume of infarcted myocardium (cm^3^). The infarct volume was calculated as the product of the slice thickness (cm) and the area of hyperenhanced pixels (cm^2^) with a signal intensity above the infarction threshold defined as >8 SD above the mean intensity of non-affected remote myocardium. Microvascular obstruction was defined as hypointense regions in the core of the infarction which had signal intensity less than the threshold for infarction. These regions were manually included in the infarct volume. The volume of microvascular obstruction (cm^3^) was calculated as the difference between the infarct volume before and after manual inclusion of regions of microvascular obstruction. Furthermore, the size of microvascular obstruction was expressed as percent of the total infarct volume. Ultimately, the infarct size was expressed as percent of left ventricular myocardium.

One animal in the normothermia group suffered from severe bradycardia which required manual open chest cardiac compression in order to secure the adequate circulation of the second injection of contrast media. The remote myocardium was somewhat increased in signal intensity and the threshold for infarction in this animal was therefore defined as pixels which were 3SD above the remote myocardium as determined by visual assessment. Finally, infarct size was expressed as a percentage of the area at risk (IS/AAR) in order to adjust for any difference in area at risk between the groups [27,28].

### Patchiness index

Infarct homogeneity was assessed by a patchiness index based on infarct surface area. The high resolution *ex vivo *MR images allowed quantification of the surface area of the infarct. Infarct surface area (cm^2^) was automatically determined as the product of the slice thickness (cm) and the distance along the pixel border between infarcted and non-infarcted pixels (cm) in each slice. For equally homogeneous infarcts, a larger infarct volume will yield a larger surface area. A dimensionless patchiness index was therefore calculated as the infarct surface area (cm^2^) to the power of 3/2, divided by the infarct volume (cm^3^) Thus, the patchiness index provided a method for estimating the homogeneity of the myocardial infarction adjusted for infarct size.

### Calculation and statistics

Calculations and statistics were performed using the GraphPad Prism 4.0 software. Values are presented as mean ± SEM. Statistical significance was accepted when *P *< 0.05 Non-parametric ANOVA (Kruskal-Wallis test) followed by Dunn's post test was used.

## Results

One pig in the pre-reperfusion hypothermia group died of intractable ventricular fibrillation shortly after initiation of ischemia. One pig in the post-reperfusion hypothermia group died of pulseless electrical activity 30 min after initiation of ischemia. One pig in the normothermia group died of pulseless electrical activity 15 min after initiation of ischemia. Thus, seven pigs in the pre-reperfusion hypothermia group, seven pigs in the post-reperfusion hypothermia group, and five pigs in the normothermia group were available for analysis.

### Temperature measurements

**Figure 2:**
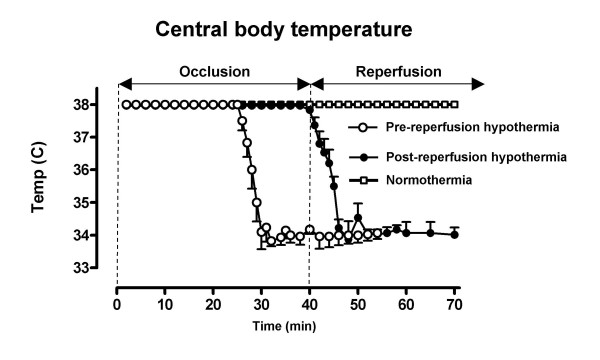
**Core body temperature (esophageal) measurements in the different groups.** The combination of infusion of cold saline with endovascular cooling caused a rapid reduction in core body temperature. Data are expressed as mean ± SEM.

Measurements of core temperature during the experiment are shown in figure 2. At the time of balloon inflation there was no difference in temperature between the groups. In approximately five minutes after initiation of hypothermia, the temperature had been lowered to below 35.0°C in all animals. There was a significant difference in temperature at the time of reperfusion between the hypothermia groups (pre-reperfusion hypothermia: 34.2 ± 0.4°C; post-reperfusion hypothermia: 37.8 ± 0.2°C; p < 0.001).

### Arrhytmias

The occurrence of ventricular tachycardia/fibrillation during ischemia and at the onset of reperfusion was recorded. VT/VF occurred in 6/7 pigs in the pre-reperfusion hypothermia group, in 3/7 pigs in the post-reperfusion hypothermia group and in 3/5 pigs in the normothermic group.

### Heart rate, cardiac output and stroke volume

In-vivo MRI was performed on all pigs for measurement of functional data after ischemia. MRI was performed ~45 min before removal of the heart for subsequent analysis of infarct size. In 7 pigs a baseline MRI was performed 2 h before induction of ischemia. Heart rate was 63 ± 6 bpm at baseline (no difference between groups). At the time of MRI the heart rates were: 76 ± 7 bpm (pre-reperfusion hypothermia), 93 ± 8 bpm (post-reperfusion hypothermia) and 118 ± 12 bpm (normothermia). Stroke volume was 41 ± 1 ml at baseline (no difference between groups). At the time of MRI, stroke volumes were: 24 ± 3 ml (pre-reperfusion hypothermia), 25± 3 ml (post-reperfusion hypothermia) and 24 ± 5 ml (normothermia). Cardiac output was 2.5 ± 0.2 l/min at baseline (no difference between groups). At the time of MRI cardiac output was: 1.8 ± 0.2 l/min (pre-reperfusion hypothermia), 2.2 ± 0.2 l/min (post-reperfusion hypothermia) and 2.7 ± 0.5 l/min (normothermia). There was no significant difference in cardiac output or stroke volume between the different groups (p = 0.076).

### Area at risk and infarct size

**Figure 3:**
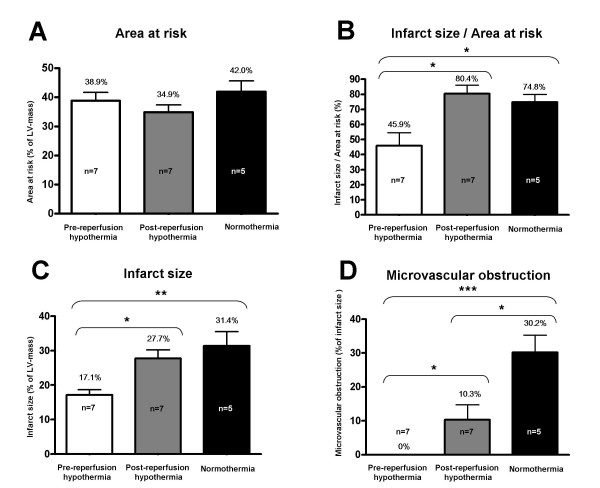
**(a) Size of area at risk (AAR) by SPECT.** There was no difference in AAR between the different groups. (b) Infarct size (IS) measured by ex-vivo MRI as a percentage of area at risk (AAR) by SPECT in the two groups. Pre-reperfusion hypothermia causes a 43% relative reduction in infarct size compared to post-reperfusion hypothermia and by 39% compared to normothermia. (c) Infarct size (IS) measured by *ex vivo *MRI, expressed as a percentage of the left ventricular mass. (d) Microvascular obstruction measured by *ex vivo *MRI, expressed as a percentage of the infarct size. Pre-reperfusion hypothermia totally abolished microvascular obstruction. Post-reperfusion hypothermia significantly decreased the extent of micovascular obstruction compared to normothermia. (* = p < 0.05, ** = p < 0.01, *** = p < 0.001). Data are expressed as mean ± SEM.

The heart was removed 4 h 22 min ± 47 min after initiation of reperfusion. There was no difference in removal time between the different groups (p = 0.22). As shown in figure 3a there was no difference in size of area at risk (AAR) between the groups (pre-reperfusion hypothermia: 39 ± 3%, post-reperfusion hypothermia: 35 ± 3%, normothermia: 42 ± 4%, (p = 0.26). Hypothermia treatment caused a significant reduction in relative infarct size (IS/AAR), (p = 0.02) between the different groups, pre-reperfusion hypothermia (46 ± 8%), post-reperfusion hypothermia (80 ± 6%), and normothermic controls (75 ± 5), (Figure 3b). The relative reduction in IS/AAR was 39% between pre-reperfusion hypothermia and normothemic controls and by 43% between pre-reperfusion hypothermia and post-reperfusion hypothermia. There was no significant difference in IS/AAR between post-reperfusion hypothermia and normothermia (p > 0.05). The infarct volume as percent of left ventricular volume (uncorrected for AAR) also differed markedly (p = 0.001): pre-reperfusion hypothermia: 17.1 ± 2% (% of left ventricle) compared to post-reperfusion hypothermia 27.7 ± 3% (p < 0.05), and normothermia 31.4 ± 4% (p < 0.01), (Figure 3c). Furthermore, 6 out of 7 pigs in the post-reperfusion hypothermia group and all 5 pigs in the normothermia group had hypointense zones in the infarction, typical for microvascular obstruction. None of the 7 pigs in the pre-reperfusion hypothermia group displayed microvascular obstruction (Figure 3d). The difference in size of the regions of microvascular obstruction was also significant (p < 0.001): Pre-reperfusion hypothermia, 0% compared to post-reperfusion hypothermia (10.3 ± 5%; p < 0.05), pre-reperfusion hypothermia compared to normothermia: (30.2 ± 5%; p < 0.001), but also between post-reperfusion hypothermia and normothermia (p < 0.05).

### Patchiness

**Figure 4:**
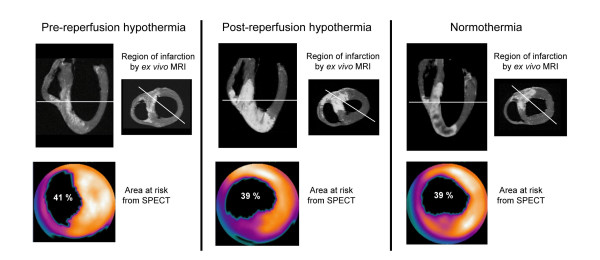
**This figure illustrates the visual comparison between typical examples from the respective groups.** The bottom row shows the area at risk measured by SPECT shown in a bull' eye plot of the left ventricle. The top row illustrates the region of infarction from *ex vivo *MRI. White lines denote the slice position of the two *ex vivo *MRI slices in relation to each other. Note the patchy pattern of the myocardial infarction in the MRI image from the pre-reperfusion hypothermia group. In the MRI images from the post-reperfusion hypothermia group and most notably, the normothermia group, hypointense zones of microvascular obstruction are seen within the area of infarction.

In the pre-reperfusion hypothermia group a patchy appearance of the myocardial infarctions was observed (Figure 4). In contrast, in the post-reperfusion hypothermia and normothermia groups the infarctions were more homogeneous in appearance. There was a significant difference in the previously described patchiness index between the groups (p = 0.002): pre-reperfusion hypothermia (241.8 ± 50.6) compared to compared to normothermia (74.3 ± 5.1; p < 0.01). There was no significant difference in patchiness index between pre-reperfusion hypothermia and post-reperfusion hypothermia (104.7 ± 5.5; p > 0.05), or between post-reperfusion hypothermia and normothermia (p > 0.05).

## Discussion

This study demonstrates that a combination of cold saline and endovascular cooling achieves a rapid induction of hypothermia which before reperfusion reduces myocardial infarct size in pigs by 43% compared to hypothermia immediately at the onset of reperfusion, and by 39% compared to normothermia. The infarctions were patchier in appearance with scattered islands of viable myocardium. For the first time we demonstrate that hypothermia at the onset of reperfusion reduces microvascular obstruction without reducing myocardial infarct size.

### Catheter based closed chest pig infarction model

All animal models have advantages and limitations. Several previous studies have used open chest models which cause a prominent operation-induced stress reaction and change the physiological situation markedly. A percutaneous catheter-based approach was chosen in order to induce ischemia with minimum trauma. The ischemia time of 40 min is shorter than in the typical patient with myocardial infarction with usually at least 2–4 hours duration before start of treatment. However, healthy pigs develop myocardial infarction more rapidly than humans [27-29], and a longer duration of ischemia would result in a large established infarct before hypothermia and reperfusion. In order to avoid a spontaneous variation in temperature during the experiment, normal core body temperature in pig (38°C) was established before induction of ischemia. In subgroup analysis of clinical trials a pre-reperfusion temperature of <35°C was sufficient to reduce infarction size by approximately 40% [16,17]. Based on the results, 33°C was chosen as target temperature, and the limit for successful hypothermia was 35°. The difference in temperature between the hypothermia groups was 3.6°C (37.8°C vs. 34.2°C) at the time of reperfusion. Thus, a reduction of core body temperature of 3.6°C before reperfusion was enough to reduce infarct size by 43%. These results are in agreement with previous studies with more long-term hypothermia during ischemia in which reductions in temperature of 3–5°C had prominent effects [8-14].

### Rapid cooling before reperfusion

One of the limitations of previous experimental studies is that animals have been subject to hypothermia before or early after induction of ischemia. In the clinical setting this is not applicable since it will only be possible to induce hypothermia shortly before or after reperfusion. Our findings are in agreement with a study by Maeng et al in which post-reperfusion hypothermia did not reduce infarct size [30]. In contrast to our findings, Hale and co-workers did not see any effect of local cooling just before reperfusion on infarct size [10]. They cooled during the final 5 min of 30 min ischemia, while we achieved = 35°C during the final 10 min of 40 min ischemia. These time differences may partly explain the difference in effect. They used topical cooling in an open chest model in rabbits. It is possible that rapid cooling from within using cold fluids combined with an intravenous catheter is more efficient. It cools primarily the most vulnerable part, the endocardium. First, cold blood passes through the right ventricle and the septum is cooled, then the whole left ventricle is cooled from the inside and last via the coronary circulation. Coronary circulation cooling is probably the least important because the artery to the affected vessel is occluded, limiting access to hypothermic fluids to the ischemic part of the myocardium.

The observed reduction in infarct size may be attributed to the protective effects of hypothermia during the last part of the ischemia. Hypothetically, it may also be an effect of a reduction in reperfusion injury. Our group has previously shown that hypothermia reduces postischemic coronary reactive hyperemia [31]. Regardless of the mechanism, the protective properties of hypothermia demonstrated in this study and previous studies indicate a potential clinical benefit of hypothermia. A major advantage of this protocol is the feasibility of "kick-starting" the hypothermia treatment using cold saline which achieves a quick cooling to target temperature. Secondly, cold saline is a low tech solution which could be administered in the ambulance, thereby inducing hypothermia before the patient reaches the cath-lab, which would further limit the normothermic ischemic time.

Post hoc analysis of the COOL-MI and ICE-IT trials demonstrated that only those patients who reached target temperature before reperfusion displayed a benefit of hypothermia [16,17]. These results suggest the necessity of inducing hypothermia before reperfusion. However, with endovascular cooling alone it takes 30 min to 1 hour to reach target temperature with current cooling methods [8,18-20]. In the clinical setting today it is not feasible to delay reperfusion therapy in order to wait for induction of hypothermia. The combination of an infusion of cold saline solution and endovascular cooling achieved a reduction in temperature to <35°C in approximately five min (Fig 2). This protocol could be clinically applicable since it would allow induction of hypothermia before or during angiography and have the patient cooled to below 35°C without delaying reperfusion therapy.

### Infarct size evaluation

Previous studies have used histology with TTC or imaging with SPECT to visualise the extent of myocardial infarction. *Ex vivo *MR imaging has been shown to correlate closely to TTC-staining in the setting of acute myocardial infarction with reperfusion for either six hours or one day [25,32]. Since *ex vivo *MRI allows higher spatial resolution (~200 images/heart) compared to TTC-staining, MRI was chosen as the method for evaluation of infarct size in our study. SPECT was used to determine area at risk during ischemia. As shown in figure 3, the placement of the balloon after the first diagonal branch induced ischemia in, on average 39% of the left ventricle, with no difference in area at risk between the groups. *Ex vivo *MRI demonstrated a more inhomogeneous and patchy appearance of the myocardial infarctions after pre-reperfusion hypothermia. Dae et al described similar findings with scattered islands of reduced Sestamibi uptake in pig hearts when assessing infarct size with SPECT in pigs subject to endovascular hypothermia [8]. It is possible that preservation of small zones of viable myocardium could be beneficial in the long term by preventing aneurysm formation and by making it possible to develop cellular hypertrophy which could improve contractility in the affected area.

### Microvascular injury

Reperfusion therapy that achieves a good angiographic result after opening of the coronary occlusion may yet result in persistent ST-segment elevation attributed to microvascular injury [33]. The degree of microvascular injury is associated with the duration of myocardial ischemia and the extent of myocardial infarction but may possibly also be caused by reperfusion injury. Importantly, the presence of microvascular obstruction is associated with a worse clinical outcome [6]. The ultrastructural alterations associated with microvascular obstruction consists of swollen endothelium with intraluminal protrusions, tightly packed erythrocytes and increased neutrophile adherence [34,35]. In our study, induction of hypothermia shortly before reperfusion abolished microvascular obstruction while it was prevalent in both hypothermia at the onset of reperfusion and the normothermia groups (Fig 3). Interestingly, there was a significant reduction in the size of microvascular obstruction when hypothermia was initiated at the onset of reperfusion compared to normothermia, possibly due to the rapid cooling by cold saline. The post-reperfusion cooling was so rapid that it actually cools during a part of the reperfusion period. The duration of this period is difficult to define, but the reactive hyperemia lasts for ten minutes, and we reached target temperature already after five minutes. Possibly, the reduction in postischemic reactive hyperemia could be a factor affecting microvascular obstruction. We recently demonstrated that hypothermia reduces reactive hyperemia during reperfusion [31]. Together these results suggest that microvascular obstruction is associated with reperfusion injury and that hypothermia may prevent reperfusion injury.

Interestingly, the reduction in microvascular obstruction in the post-reperfusion group was not accompanied by a reduction in infarct size (Figure 3). Previous studies have not been able to separate these events [36]. For example, the protective effects of both ischemic preconditioning and Na+/H+-exchanger inhibition reduces necrosis and microvascular obstruction to the same extent [36]. However, Hale et al demonstrated that hypothermia during ischemia reduced infarct size with more than proportional microvascular protection [15]. Here we support their findings and demonstrate for the first time that microvascular obstruction can be reduced without affecting infarct size when hypothermia is induced at the onset of reperfusion. This suggests separate mechanisms for the developement of microvascular obstruction and infarct size. Further studies in this model may be able to dissociate the mechanisms of microvascular obstruction from myocardial infarction development.

### Hemodynamic measurements

There was a trend towards a lower cardiac output in the pre-reperfusion hypothermia group after myocardial infarction was induced. The stroke volume remained unchanged, thus any difference in cardiac output would be attributed to a difference in heart rate. We did not measure the core temperature when the MRI was performed. Possibly, the temperature in the pre-reperfusion hypothermia group could be lower, accounting for the difference in heart rate. We did not expect to see any acute improvement in left ventricular function in the hypothermia groups since the area at risk between the groups did not differ, thus the different groups would have had the same amount of myocardium subject to ischemia. The benefit in a lower infarct size would be expected during the time course of days-weeks as the stunned myocardium would regain function and ventricular remodelling would be prevented.

## Limitations

The methods for ex vivo quantification of patchiness index and microvascular obstruction are novel methods that have not been validated as such, however we have previously shown that ex vivo contrast enhanced T1 weighted MRI, as used in our study, shows excellent agreement with in vivo delayed enhancement MRI [37]. Algorithms similar to that used for patchiness index in this study have previously been used for quantification and characterization of myocardial infarction using delayed enhancement MR imaging [38]. Demonstration, quantification and validation of microvascular obstruction in MR imaging have been performed with microspheres in experimental models [39] and extensively studied in humans using both first-pass MR perfusion imaging and delayed contrast enhanced MR imaging.

## Conclusion

A rapid induction of hypothermia can be achieved by a combination of cold saline and endovascular cooling. Such hypothermia induced before reperfusion is effective in reducing the myocardial infarct size and protecting the heart from microvascular obstruction. Interestingly, rapid induction of hypothermia at the onset of reperfusion also reduced microvascular obstruction with no effect on infarct size. These effects may have significant beneficial effects on clinical outcome after myocardial infarction. This protocol can easily be applied to the clinical setting where hypothermia could be induced before reperfusion without delaying PCI. However, a rapid infusion of large volumes of saline solution in patients with myocardial infarction could have serious side-effects, such as left ventricular overload and pulmonary oedema. A currently ongoing human safety and feasibility study on patients with acute myocardial infarction will determine whether cold saline as an adjunctive therapy to endovascular cooling can be safely administered (RAPID MI-ICE pilot) [40].

## Abbreviations

AAR Area at risk

IS Infarct size

SPECT Single photon emission computed tomography

MRI Magnetic resonance imaging

LAD Left anterior descending artery

PCI Percutaneous coronary intervention

## Competing interests

The author(s) declare that they have no competing interests.

## Authors' contributions

MG conceived, coordinated and carried out animal experiment, analyzed data, performed statistical analysis, drafted and wrote the manuscript. GKO participated in the animal experiment, carried out lab analysis and also gave invaluable advice on the manuscript. HE and MU designed and performed the MRI data acquisition and image analysis, and gave invaluable advice on both the animal experiments and the manuscript. JVDP participated in the animal experiments and gave invaluable advice on the manuscript. EH developed the novel functionality for the MRI analysis software and gave invaluable advice on the manuscript. HA conceived the method to normalize final infarct size as measured by delayed enhancement MR imaging to area at risk as measured by myocardial perfusion SPECT imaging and gave invaluable advice on both the animal experiment and the manuscript. DE conceived the study, participated in the animal experiment and wrote the manuscript. All authors read and approved the final manuscript.

## Pre-publication history

The pre-publication history for this paper can be accessed here:


